# *Candida *esophageal perforation and esophagopleural fistula: a case report

**DOI:** 10.1186/1752-1947-2-209

**Published:** 2008-06-17

**Authors:** Baha Al-Shawwa, Lynn D'Andrea, Diana Quintero

**Affiliations:** 1 Department of Pediatrics, Medical College of Wisconsin (Pulmonary Section), Children's Hospital of Wisconsin, West Wisconsin Avenue, Milwaukee, WI53226, USA

## Abstract

**Introduction:**

Esophageal perforation is a rare disease, which can lead to significant morbidity and mortality. Its clinical presentation can mimic other disease processes and, therefore, it can be easily misdiagnosed. *Candida *infection of the esophagus is an extremely rare cause of esophageal perforation.

**Case presentation:**

We report the youngest pediatric case in the medical literature of spontaneous esophageal perforation and an esophagopleural fistula due to *Candida *infection.

**Conclusion:**

A high index of suspicion, especially in the presence of *Candida *empyema and the absence of disseminated infection, should raise the possibility of esophageal perforation with esophagopleural fistula formation. This can lead to early diagnosis and surgical intervention, which would decrease the high mortality rate of this rare condition.

## Introduction

Esophageal perforation is a rare and usually life-threatening disease, especially in children. A delay in diagnosis and management worsens the outcome and increases the risk of complications [1]. Esophageal perforation usually occurs with the use of endoscopic instruments, or in relation to surgical thoracic procedures, trauma or foreign bodies. Spontaneous esophageal rupture rarely occurs unless it is associated with forceful episodes of vomiting (Boerhaave syndrome) [2].

Esophegeal perforation should be suspected on the basis of clinical presentation of sudden chest pain, fever, vomiting and subcutaneous emphysema. However, in children the presentation of esophageal perforation can mimic many disease processes, such as pneumonia, lung abscess and sepsis, especially in patients with multiple medical problems. Therefore, a high index of suspicion is required [3].

In this case report we present a patient with a spontaneous esophageal perforation that was associated with *Candida *infection and complicated by an esophagopleural fistula (EPF).

## Case presentation

The patient was a 7-year-old boy with a complex medical history including prematurity, as well as holoprosencephaly, congenital absence of the corpus callosum and hydrocephalus. A shunt malfunction at 6 years of age left him with severe neurological impairment. After this event, he required a tracheotomy for long-term ventilatory support and a gastrostomy tube for nutritional support. He was also being treated for gastro-esophageal reflux disease.

He presented to the emergency room (ER) with a day's history of fever, difficulty breathing and decreased urine output. He was severely hypoxic (SpO_2 _in the 50s on room air) and had poor perfusion. He was resuscitated in the ER and was admitted to the intensive care unit with a diagnosis of respiratory failure and presumed sepsis. Initial evaluation revealed an elevated white blood cell count at 32,800 with 50% left shift and severe metabolic and respiratory acidosis (pH 6.96, PCO2 77, HCO3 16.5 and base deficit of 17.2). Chest X-ray showed bilateral pneumonia and large pleural effusions. He had bilateral chest tubes placed with return of purulent, exudative pleural fluid. He was started on broad-spectrum antibiotics, including cefotaxime and vancomycin, as well as inotropic support. Lysosomal amphotericin B was added on day 3 when the pleural fluid culture was positive only for *Candida albicans*. Blood and urine cultures remained negative.

**Figure 1:**
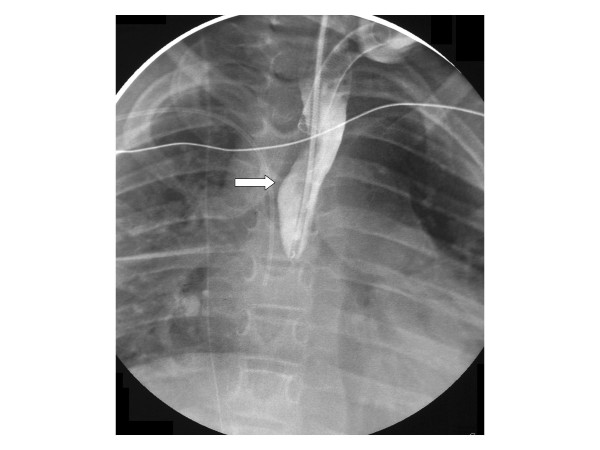
**Proximal esophagus with blind pouch**. A catheter is present for contrast.

**Figure 2:**
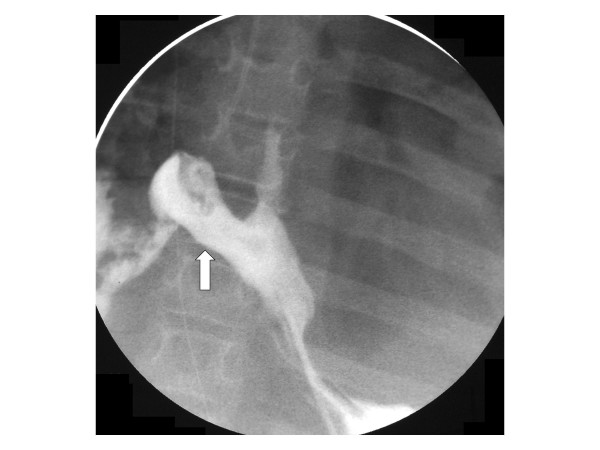
**Distal esophageal pleural fistula**. Under fluoroscopic guidance, a catheter was placed in the distal esophagus through a gastrostomy tube.

The patient's clinical condition improved quickly and he was off inotropic support in 2 days and back to his home ventilator setting in 3 days. The left chest tube was removed on day 6, but he continued to have persistent right chest tube drainage and positive culture with *C. albicans *for 2 weeks. Extensive humeral and cellular immunological testing and infectious disease evaluation including cultures and radiological testing revealed no evidence of a disseminated *Candida *infection or underlying immunodeficiency. The diagnosis of an esophagopleural fistula (EPF) was considered and upper gastrointestinal studies confirmed this suspicion (Figures 1 and 2). The patient underwent surgical intervention and was found to have frank esophageal perforation, a chronic right empyema, a diffuse abscess cavity in the right chest and an intense inflammatory process likely due to *Candida *infection, which had been isolated from the pleural fluid immediately after hospitalization. Esophagectomy with cervical esophagostomy were performed and owing to his permanent disability, reconstruction of alimentary continuity was deferred.

## Discussion

*Candida *colonization of the esophagus occurs in 25% of healthy individuals [4]. However, invasive *Candida *esophageal infections predominantly occur in immunocompromised and transplant patients or after a major surgical procedure [5]. This is a case report of the youngest reported pediatric patient with a spontaneous lower esophageal perforation due to *Candida *infection, and which led to the formation of an EPF.

There have been six previous reported cases of esophageal perforation associated with *Candida *infection, however, most of these were in immunocompromised patients. Jones et al. [3] reported two fatal cases of severe necrotizing *Candida *esophagitis in diabetic patients with renal transplantations. Another two non-fatal cases were reported by Gaissert et al. [4]; one with underlying leukemia and the other after esophageal instrumentation. Also, Gock et al. [5] reported a 76-year-old immunocompromised woman who had a paraesophageal hernia. Abildgaard et al. [6] reported a total expulsion of the distal esophagus due to invasive *Candida *esophagitis in a 30-year-old with acute leukemia.

In our case, the patient was not immunocompromised and had no instrumentation or surgical interventions for over a year before presentation. He did, however, have long-standing gastro-esophageal reflux, which probably caused mucosal damage at the gastro-esophageal junction. The *Candida *esophagitis was probably facilitated by the damaged mucosa.

## Conclusion

The clinical presentation of esophageal perforation can mimic other processes such as aspiration pneumonia and lung abscess, especially in a pediatric patient with a complex medical history as in this reported case. Therefore, a high index of suspicion, especially in the presence of *Candida *empyema and the absence of disseminated infection, should raise the possibility of esophageal perforation with EPF formation. This can lead to early diagnosis and early surgical intervention and treatment, which can decrease the high mortality in this rare and serious condition.

## Competing interests

The authors declare that they have no competing interests.

## Authors' contributions

BA collected the data and drafted the manuscript, LD, DQ and BA participated in writing, revising and approving the final manuscript.

## Consent

Written informed consent was obtained from the patient's next-of-kin for publication of this case report and any accompanying images. A copy of the written consent is available for review by the Editor-in-Chief of this journal.
